# Prenatal exposure to multiple metallic and metalloid trace elements and the risk of bacterial sepsis in extremely low gestational age newborns: A prospective cohort study

**DOI:** 10.3389/fepid.2022.958389

**Published:** 2022-09-07

**Authors:** Catherine M. Bulka, Lauren A. Eaves, Amaree J. Gardner, Patrick J. Parsons, Aubrey L. Galusha, Kyle R. Roell, Lisa Smeester, T. Michael O’Shea, Rebecca C. Fry

**Affiliations:** 1 Department of Environmental Sciences and Engineering, Gillings School of Global Public Health, University of North Carolina at Chapel Hill, Chapel Hill, NC, United States,; 2 Division of Environmental Health Sciences, Wadsworth Center, New York State Department of Health, Albany, NY, United States,; 3 Department of Environmental Health Sciences, School of Public Health, University at Albany, Rensselaer, NY, United States,; 4 Institute for Environmental Health Solutions, Gillings School of Global Public Health, University of North Carolina at Chapel Hill, Chapel Hill, NC, United States,; 5 Department of Pediatrics, School of Medicine, University of North Carolina, Chapel Hill, NC, United States,; 6 Curriculum in Toxicology and Environmental Medicine, University of North Carolina at Chapel Hill, Chapel Hill, NC, United States

**Keywords:** metals, mixtures, pregnancy, sepsis, preterm (birth)

## Abstract

**Background:**

Prenatal exposures to metallic and metalloid trace elements have been linked to altered immune function in animal studies, but few epidemiologic studies have investigated immunological effects in humans. We evaluated the risk of bacterial sepsis (an extreme immune response to bacterial infection) in relation to prenatal metal/metalloid exposures, individually and jointly, within a US-based cohort of infants born extremely preterm.

**Methods:**

We analyzed data from 269 participants in the US-based ELGAN cohort, which enrolled infants delivered at <28 weeks’ gestation (2002–2004). Concentrations of 8 trace elements—including 4 non-essential and 4 essential—were measured using inductively coupled plasma tandem mass spectrometry in umbilical cord tissue, reflecting *in utero* fetal exposures. The infants were followed from birth to postnatal day 28 with bacterial blood culture results reported weekly to detect sepsis. Discrete-time hazard and quantile g-computation models were fit to estimate associations for individual trace elements and their mixtures with sepsis incidence.

**Results:**

Approximately 30% of the extremely preterm infants developed sepsis during the follow-up period (median follow-up: 2 weeks). After adjustment for potential confounders, no trace element was individually associated with sepsis risk. However, there was some evidence of a non-monotonic relationship for cadmium, with hazard ratios (HRs) for the second, third, and fourth (highest) quartiles being 1.13 (95% CI: 0.51–2.54), 1.94 (95% CI: 0.87–4.32), and 1.88 (95% CI: 0.90–3.93), respectively. The HRs for a quartile increase in concentrations of all 8 elements, all 4 non-essential elements, and all 4 essential elements were 0.92 (95% CI: 0.68–1.25), 1.19 (95% CI: 0.92–1.55), and 0.77 (95% CI: 0.57–1.06). Cadmium had the greatest positive contribution whereas arsenic, copper, and selenium had the greatest negative contributions to the mixture associations.

**Conclusions:**

We found some evidence that greater prenatal exposure to cadmium was associated with an increased the risk of bacterial sepsis in extremely preterm infants. However, this risk was counteracted by a combination of arsenic, copper, and selenium. Future studies are needed to confirm these findings and to evaluate the potential for nutritional interventions to prevent sepsis in high-risk infants.

## Background

Sepsis, an extreme immune response to an infection of the bloodstream, is a leading cause of neonatal morbidity and mortality. In the last decade, there were an estimated 3,930 cases of neonatal sepsis per 100,000 live births worldwide and nearly 20% of cases were fatal (1). While proximal risk factors such as preterm labor, prolonged rupture of membranes, and intra-amniotic infections have been recognized (2), preventing neonatal sepsis remains a key challenge (3). Identifying more distal risk factors for neonatal sepsis that are modifiable may be a promising strategy, as it is often too late to prevent fetal and neonatal infections by the time medical attention is sought (3). One such plausible risk factor is prenatal exposure to environmental metallic and metalloid trace elements, which are ubiquitous in the air, soil, water, and food supply (4). Several non-essential trace elements, including arsenic (a metalloid) (5), cadmium (a metal) (6), lead (a metal) (7), and mercury (a metal) (8), are recognized as immunotoxicants. In contrast, other nutritionally-essential trace elements such as copper (a metal) (9), manganese (a metal) (10), selenium (a metalloid) (11), and zinc (a metal) (10) may be necessary for optimal immune function, with experimental studies in animals indicating adverse effects from deficiencies and excesses (12–15).

Non-essential metallic and metalloid trace elements have distinct mechanisms of immunotoxicity, with many implicated as immunosuppressants, particularly when exposures occur early in life. For example, arsenic exposures in childhood have been linked to reductions in the proportion of CD4 (“helper”) T cells in peripheral blood (16), which capture and kill bacteria from infected dendritic cells. Prenatal exposures to cadmium have also been tied to alterations in lymphocyte composition in infancy (17), and prenatal methylmercury exposures to alterations that persist into childhood (18). At the same time, non-essential metals and metalloids could promote inflammation, which is an appropriate response to the presence of infection but can be dangerous when excessive (19). Research on lead has identified links between *in utero* exposures with lower concentrations of the anti-inflammatory cytokines interleukin (IL)-4 and IL-8, as well as higher concentrations of the pro-inflammatory cytokine tumor necrosis factor alpha (TNF-α) in early childhood (20), suggesting exposures may have implications for infection elimination and/or tissue damage (19). In the United States, over 85% of women of reproductive age have measurable levels of arsenic, cadmium, lead, and mercury in their bodies (21), making immunotoxic effects of prenatal exposures a pertinent concern.

It has been posited that non-essential metals and metalloids gain entry into human cells by “mimicking” and potentially competing with nutritionally-essential trace elements (22). For this reason, epidemiologic studies of exposure mixtures encompassing both non- and nutritionally-essential trace elements have become popular under the premise that nutritional interventions could counteract environmental metal/metalloid toxicity (23). Although few epidemiologic studies to date have considered the role of complex trace element mixtures in the development of immune-mediated and infectious diseases (23), nutritionally-essential elements are recognized for their important roles in the immune system. Zinc, for example, modulates the inflammatory response by binding to and inhibiting IKK-β, a protein important for cytokine-activated intracellular signaling. In a mouse model of polymicrobial sepsis, a zinc-deficient diet promoted insufficient control of IKK-β, leading to excessive inflammation (24). Therefore, we hypothesized that greater prenatal exposures to nutritionally-essential trace elements, namely copper, manganese, selenium, and zinc would be associated with a reduced risk of neonatal sepsis, whereas the opposite would be true for the non-essential trace elements arsenic, cadmium, mercury, and lead (i.e., greater prenatal exposures to non-essential trace elements would be associated with an increased risk of neonatal sepsis). To test these hypotheses, we analyzed each element individually and complemented this approach with quantile g-computation to assess how combinations of exposures *in utero* influences the risk of sepsis within the first few weeks of life. We conducted all analyses within the Extremely Low Gestational Age Newborns (ELGAN) cohort, which is comprised of infants born extremely preterm who are at a markedly increased risk of developing neonatal bacterial sepsis due to having immature immune systems and requiring invasive medical procedures and devices (25).

## Materials and methods

### The ELGAN cohort

The ELGAN study is an ongoing, multi-center, prospective cohort originally designed to assess contributors to brain damage in extremely preterm infants (born prior to 28 weeks’ gestation) (26). Infants were enrolled from 2002 to 2004 from 14 study sites across five states: Connecticut, Illinois, Massachusetts, Michigan, and North Carolina. The institutional review boards of each of the participating institutions approved enrollment and consent procedures. Of the 1,506 infants initially enrolled, umbilical cord tissue samples used to quantify prenatal metal and metalloid exposures were available for a subset of *n* = 292. However, 12 of the specimens had insufficient sample mass and another 11 infants were excluded for missing data on relevant covariates (detailed further below), bringing the final analytic sample size to *n* = 269.

### Assessment of prenatal metal and metalloid trace element exposures in umbilical cord tissue

Study pathologists collected umbilical cord tissue specimens according to a standardized protocol. Briefly, using a sterile technique and scissors, two 1-cm segments of tissue were collected from the extra-abdominal umbilical cord attached to each infant within 1-h of being clamped and cut following delivery. The segments were immediately placed into separate cryovials, immersed into liquid nitrogen, and transported to a −80°C freezer for long-term storage. The samples were later shipped on dry ice to the Wadsworth Center for quantification of metal and metalloid trace elements.

Upon receipt at the Wadsworth Center, the umbilical cord samples were checked against the shipping manifest, accessioned per standard laboratory procedure, and stored at −80°C pending analysis. Samples were later thawed and, if applicable, sectioned into segments for analysis with a final dry mass of ~50 mg. Samples were sectioned using high-purity tantalum tools that were fabricated in-house to avoid contamination with other trace elements typically found in stainless steel. All samples were then rinsed with double-deionized (DDI) water to remove superficial blood and placed in a 13-ml acid-washed tube before being freeze-dried to constant mass using a slow 5-step program to ensure thorough removal of water content. The samples were digested in concentrated double-distilled HNO_3_ using a Microwave Assisted Reaction System (MARS) 6 equipped with Xpress vessels *via* the “One-Touch Animal Tissue” method. Digests were diluted to 10-mL with DDI water and stored at 4°C pending analysis. Sample digests were analyzed using an Agilent 8900 Inductively Coupled Plasma tandem Mass Spectrometer (ICP-MS/MS) equipped with a SPS 4 autosampler and an Octopole Reaction System (ORS) with axial acceleration technology. The method was optimized for analyzing umbilical cord tissue samples while reducing polyatomic and isobaric interferences. The method was validated against four Standard Reference Materials (SRM) obtained from the National Institute of Standards and Technology (NIST), Gaitherburg, MD: NBS 1577 Bovine Liver, NIST 1577b Bovine Liver, NIST 1577c Bovine Liver, and NIST 8414 Bovine Muscle Powder. The SRMs were freeze-dried, digested, and analyzed in the same manner as the cord samples. Values for sample spikes, duplicates, blanks, and calibrators were carefully monitored throughout the study. A subset of sample digests were analyzed in duplicate (*n* = 75) or triplicate (*n* = 23) consistent with the Wadsworth Center’s quality management system; the final trace element concentration was reported as the average of the replicate values. The method limits of detection (LOD) for the 8 trace elements were: arsenic (0.42 ng/g), cadmium (0.32 ng/g), copper (0.074 μg/g), lead (2.6 ng/g), manganese (0.010 μg/g), mercury (0.79 ng/g), selenium (0.10 μg/g), and zinc (1.4 μg/g). All umbilical cord tissue samples had detectable levels of arsenic, copper, lead, manganese, selenium, and zinc. For mercury and cadmium, one and five samples respectively were at or below the detection limit so concentration values were replaced by the corresponding limit divided by √2.

### Diagnostic testing for bacterial sepsis

After delivery, trained research nurses used structured data collection forms to review medical charts. As previously described by Patel et al. (27) infant bacterial blood cultures were reported on the forms for days 7, 14, 21, and 28. As a result, the specific day on which the blood harbored an organism is unknown, only the week during which bacteremia first occurred. Incident bacterial sepsis was defined as the first instance of bacteremia.

Neonatal sepsis is often characterized by the timing of onset with cases presenting at <7 days of life referred to as early-onset and afterwards as late-onset (25). This characterization is based on suspected etiology. Early-onset cases are generally considered to be the result of pathogen transmission from the female genitourinary tract, infecting the fetus *in utero* or during delivery. In contrast, late-onset cases are hospital- or community-acquired. The bacteria responsible for the initial infection tends to differ by timing of onset as well. The majority of early-onset cases are attributable to *Escherichia coli* and group B streptococcus account whereas late-onset cases are mostly driven by Coagulase-Negative Staphylococci and *Staphylococcus aureus* (28, 29). The ELGAN study did not collect information on the bacterial species isolated from cultures (27). However, we divided sepsis cases as early- or late-onset to examine potential differences in metal/metalloid-associated risk profiles.

### Covariates

We considered adjusting for many factors that might confound associations of prenatal metal and metalloid trace element exposures with neonatal bacterial sepsis. We narrowed these down by using a directed acyclic graph (DAG) to identify variables associated with both metal/metalloid exposures and sepsis, excluding any that could be causal intermediates (Supplementary Figure 1) (30). In all models, we adjusted for the geographic region in which the infant was delivered as well as maternal race/ethnicity, age, educational attainment, Medicaid coverage (a proxy for income), pre-pregnancy body mass index (BMI), and smoking status during pregnancy. Maternal sociodemographic characteristics pre-pregnancy BMI were self-reported shortly after delivery. Pregnancy and infant characteristics were abstracted from medical records.

### Statistical analyses

All statistical analyses were conducted in R (version 4.0.3) (31).

For each trace element, we first calculated measures of central tendency and distributions, both overall and stratified by covariates. Most of the trace elements had right-skewed distributions, particularly the non-essential elements. Therefore, we calculated Spearman’s rank coefficients (ρ) to evaluate how the metals and metalloids were correlated with one another.

To conduct a time-to-event analysis of incident bacterial sepsis, which was ascertained on a weekly basis, the data were transformed into a person-period structure (32). In its original form, the data were organized such that each infant was represented by one row; after transformation, each infant had multiple rows reflecting each week they were under observation. Infants who were transferred to another hospital (and therefore lost to follow-up) or who did not develop sepsis during the first 28 days of life were right-censored.

To analyze the trace elements individually, we fit separate discrete time hazard models to estimate hazard ratios. The models were based on the probability of developing bacterial sepsis each week, conditional on being sepsis-free the week prior. Conditional probabilities were estimated using a complementary log-log link function with four baseline hazards (i.e., one for each week of follow-up time) (33). Indicator variables were used to model quartiles of cord metal/metalloid concentrations (relative to the lowest quartile) after adjustment for potential confounders. Geographic region (the Midwest, New England, or North Carolina), race/ethnicity (Hispanic, non-Hispanic black, non-Hispanic white, non-Hispanic other), educational attainment level (less than a high school diploma, high school diploma, or college degree), Medicaid coverage, and smoking status during pregnancy (yes or no) were modeled using indicator variables whereas age at delivery (years) and pre-pregnancy BMI (kg/m^2^) were modeled continuously. Because recruitment into the study was based on extremely low gestational ages and, consequently, many of the infants were multiples (e.g., twins or triplets), we computed cluster-robust standard errors *via* bootstrapping at the pregnancy level with 1,000 iterations (34). We tested for deviations from the proportional hazards assumption by using likelihood ratio tests to compare models with and without interaction terms for metals/metalloids and time. To test for linear relationships between cord concentrations and sepsis incidence, we modeled the median trace element concentration in each quartile as a continuous variable (35); we considered the corresponding *p*-value as indicative of a linear trend if it was below 0.05.

To analyze trace element mixtures, we used quantile g-computation (36). These models were fit similarly to the individual element models in that we again used a complementary log-link function with four baseline hazards and covariates to estimate adjusted discrete-time hazard ratios. However, instead of estimating hazard ratios for each metal/metalloid individually, quantile g-computation estimates a hazard ratio for the exposure mixture. This hazard ratio can be interpreted as the change in the log-hazard of sepsis per a simultaneous one-quartile increase of all elements in the mixture. We tested three sets of mixtures: (1) all 8 trace elements combined; (2) the 4 non-essential trace elements (arsenic, cadmium, lead, mercury); and (3) the 4 essential trace elements (copper, manganese, selenium, zinc). For the latter two sets, the trace elements not included in the specific exposure mixture of interest were still included as model covariates so as to control for potential confounding by co-exposures. Adjustments were also made for the same model covariates included in the individual trace element models. In each of the quantile g-computation models, the mixture components were assigned a weight that can be either negative or positive in direction. If all assigned weights are in the same direction, they are constrained to sum to one and can be interpreted as the proportional contribution of a given trace element to the overall association with incident sepsis. If the assigned weights have different directions, their absolute values are instead constrained to sum to two, constituted by one for the sum of all positive weights and one for the sum of all negative weights, and can be interpreted as the proportional contribution of a given trace element to the positive (or negative) partial association with incident sepsis. Cluster-based bootstrapping with 1,000 iterations was performed to estimate standard errors that appropriately account for the multiple pregnancies in the dataset.

### Sensitivity and stratified analyses

For both individual and trace element mixtures, we attempted to re-fit the models stratified by early- or late-onset, but the number of early-onset cases (*n* = 19) was too small for reliable estimation. Instead, we performed analyses restricted to late-onset cases to explore whether there were any differences in metal/metalloid associations from the models of sepsis overall.

Because the ELGAN cohort sampled participants born extremely preterm at <28 weeks’ gestation, our analyses were implicitly partially adjusted for gestational age. However, as depicted in our DAG (Supplementary Figure 1), gestational age was considered to be a consequence of prenatal metal and metalloid exposures (37), making it an intermediate on the hypothesized causal pathway. To get a better approximation of the *direct* effect of prenatal metals/metalloids exposure on the risk of sepsis, we re-fit models explicitly adjusting for all intermediates and their descendants: gestational age, birth weight, histologically-confirmed chorioamnionitis (38), infant sex, preterm premature rupture of membranes (pPROM), and infections during pregnancy. Infections during pregnancy was a composite variable including diagnoses of a urinary tract, vaginal, or group B streptococcus infection, or any antibiotic treatment.

Finally, since animal and epidemiologic studies of *in utero* metal and metalloid exposures have reported sex-specific associations with immunological endpoints (17, 39), we fit models stratified by infant sex. To formally test for effect measure modification between females and males, we computed two-sample *z-*test *p*-values and considered any <0.20 to be significant (40).

## Results

### Population characteristics

**FIGURE 1:**
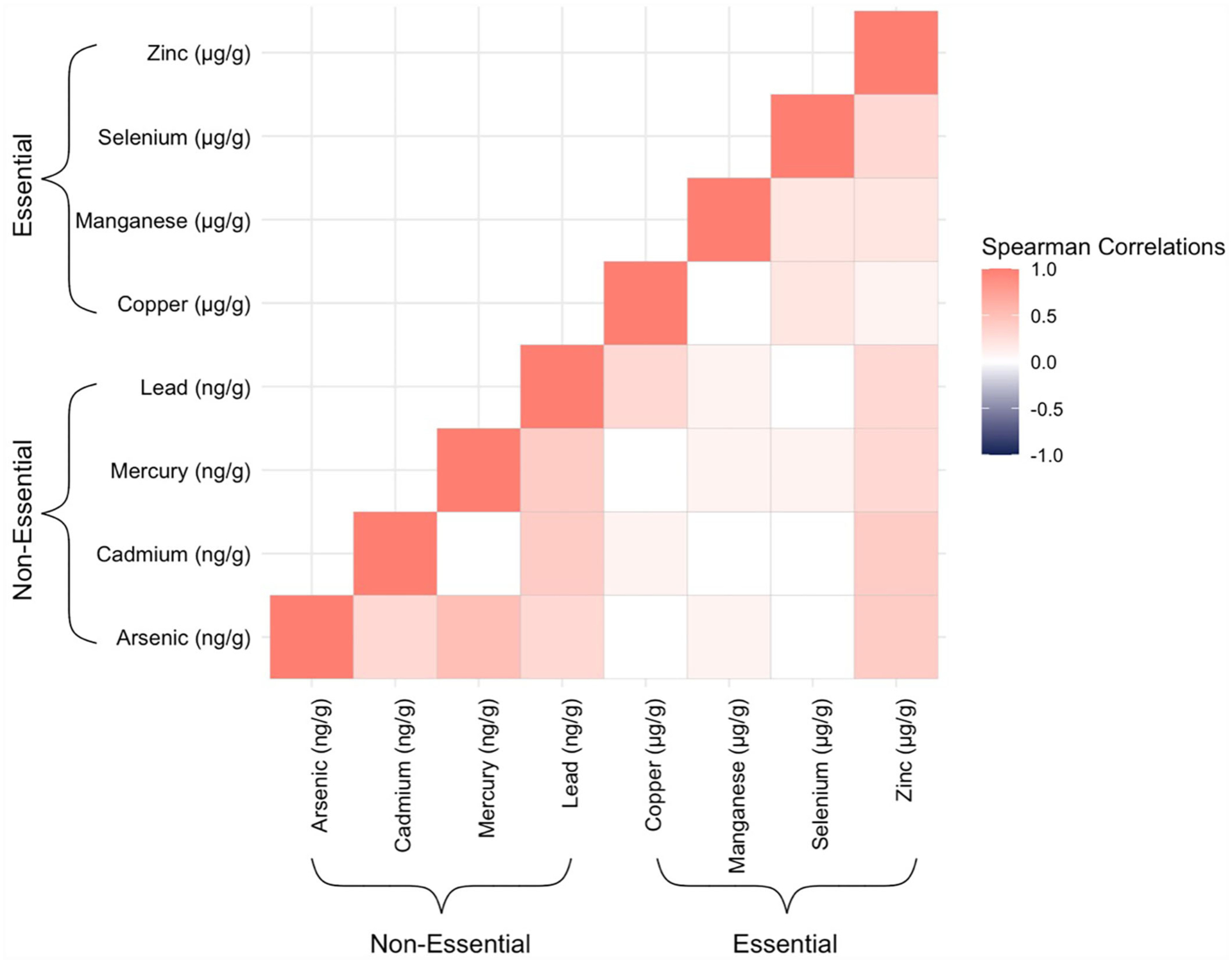
Heatmap displaying Spearman’s rank correlation coefficients between all possible pairs of the trace elements, as measured in ELGAN umbilical cord tissue samples (*n* = 269).

Mothers in our study sample were predominantly over the age of 30 years (56%), non-Hispanic white (61.7%), and not on Medicaid (68.0%) (Table 1). Cord tissue concentrations of several trace elements, notably arsenic, mercury, lead, and selenium, appeared to increase with maternal age. Differences were also observable by maternal race/ethnicity and geographic region. Hispanic mothers gave birth to infants with particularly high levels of arsenic and lead in their umbilical cords. Mothers who reported smoking during pregnancy gave birth to infants with nearly twice the amounts of cadmium in cord tissue than mothers who reported not smoking. Median concentrations of arsenic, mercury, and lead from New England-born infants were approximately double those born elsewhere. There were slightly more male infants than female (55.4/44.6%). In total, 81 (30.1%, Table 1) infants developed bacterial sepsis during the median follow-up time of 2 weeks (range: 1–4 weeks) and only 3 (1.1%, data not shown) infants were lost to follow-up because they were transferred to a non-ELGAN affiliated hospital. With respect to the trace elements, umbilical cord concentrations were mostly positively correlated with one another with the highest Spearman’s rank correlation coefficient observed for arsenic and mercury (*ρ* = 0.5) (Figure 1).

### Individual trace element models

**FIGURE 2:**
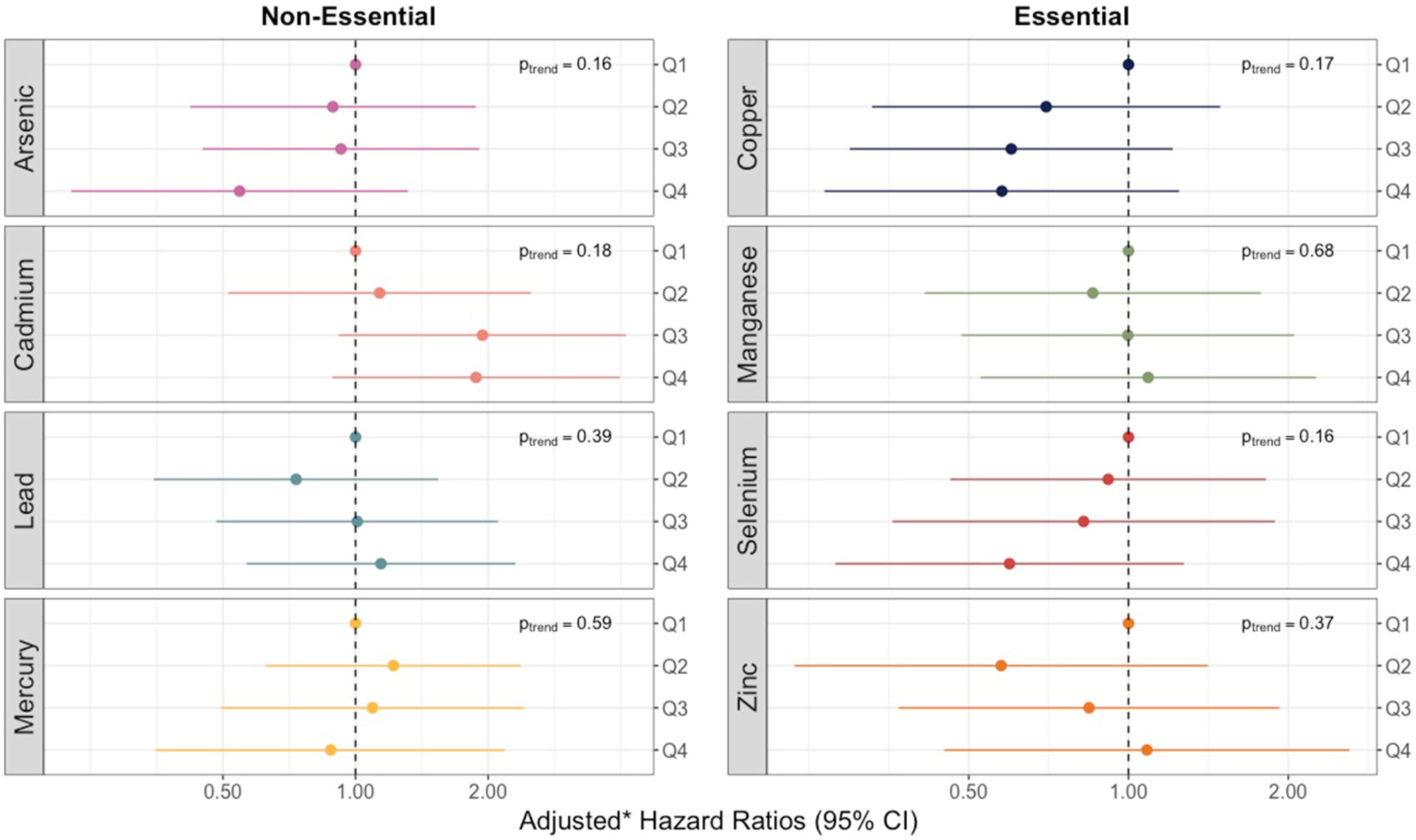
Forest plots showing adjusted* hazard ratios with 95% confidence intervals for neonatal bacterial sepsis in the first 28 days of life by trace element quartiles. *p*_trend_ for linear associations with sepsis calculated by modeling the median trace element concentration in each quartile as a continuous variable. *Adjusted for geographic region, maternal race/ethnicity, age, educational attainment, Medicaid coverage, pre-pregnancy BMI, and smoking status during pregnancy.

We observed no violations of the proportional hazards assumption for any of the 8 trace elements assessed. None of the models for individual trace elements, as measured in umbilical cord tissue, produced significant relationships or linear trends with overall sepsis incidence (Figure 2). However, hazard ratios for increasing quartiles of cadmium were markedly elevated relative to the other trace metals and showed some evidence of a non-monotonic dose-response with the risk of bacterial sepsis. Compared to the lowest quartile, the adjusted hazard ratios for cadmium concentrations in the second, third, and fourth (highest) quartiles were 1.13 (95% CI: 0.51–2.54), 1.94 (95% CI: 0.87–4.32), and 1.88 (95% CI: 0.90–3.93) (Supplementary Table 1). The hazard ratios for some of the other trace elements were noticeably below the null, including the highest quartiles of arsenic, copper, and selenium (Figure 2; Supplementary Table 1).

When focusing on late-onset sepsis, associations were generally consistent with associations for any sepsis (Supplementary Table 2), albeit less precise. Adjusting for hypothesized intermediates also did not materially change the results (Supplementary Table 3).

In sex-stratified models, associations between cadmium and sepsis were strongest for female infants (Supplementary Table 4). The adjusted hazard ratio comparing the highest to the lowest quartile was 4.54 (95% CI: 0.86–23.89) among females, but only 1.19 (95% CI: 0.35–4.03) among males. However, *p*-values for effect measure modification for cadmium and all other trace elements were above 0.20 (Supplementary Table 4).

### Trace element mixture models

**FIGURE 3:**
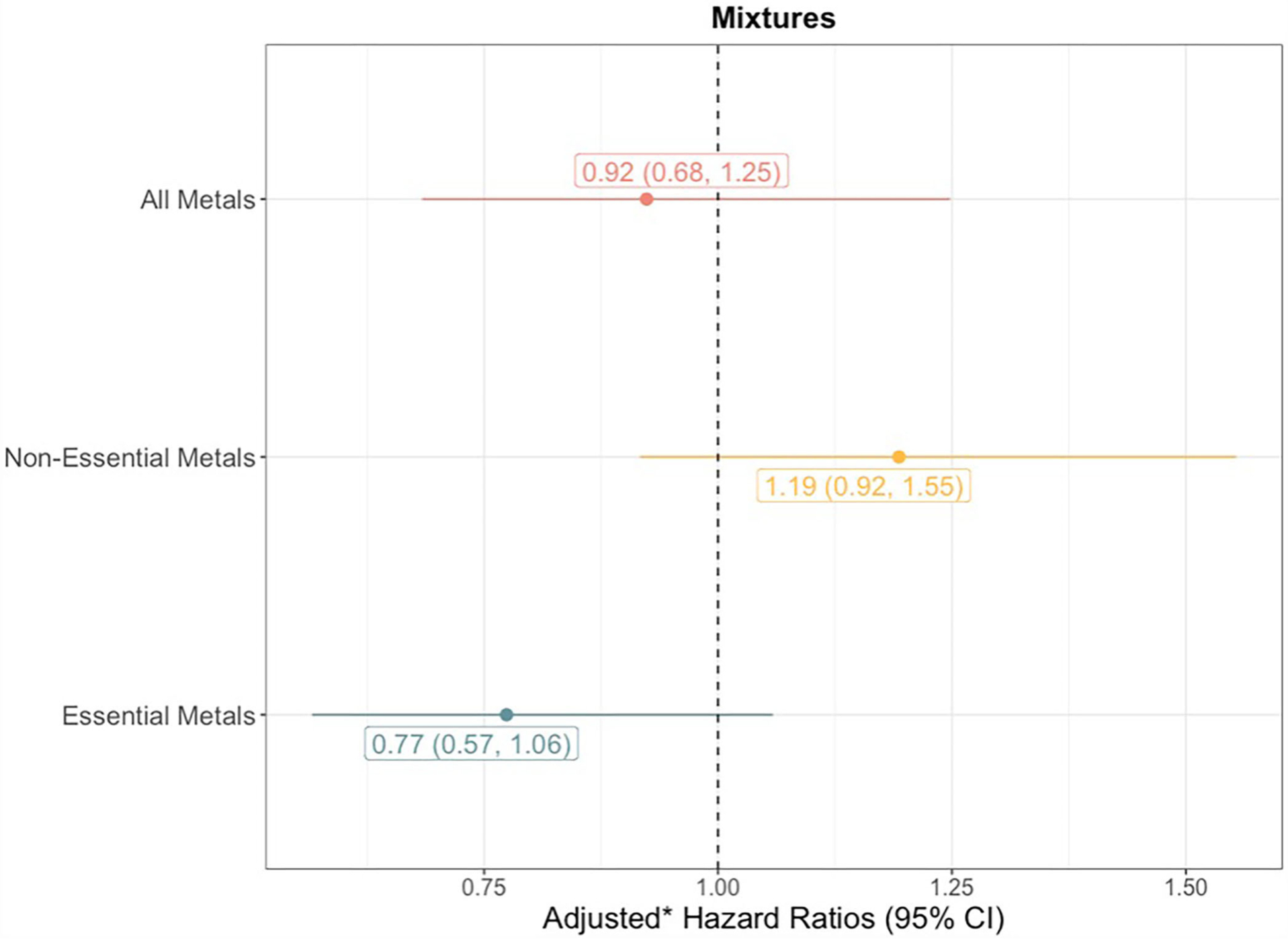
Forest plot showing adjusted* hazard ratios with 95% confidence intervals for neonatal bacterial sepsis in the first 28 days of life by trace element mixture. *Adjusted for geographic region, maternal race/ethnicity, age, educational attainment, Medicaid coverage, pre-pregnancy BMI, and smoking status during pregnancy.

**FIGURE 4:**
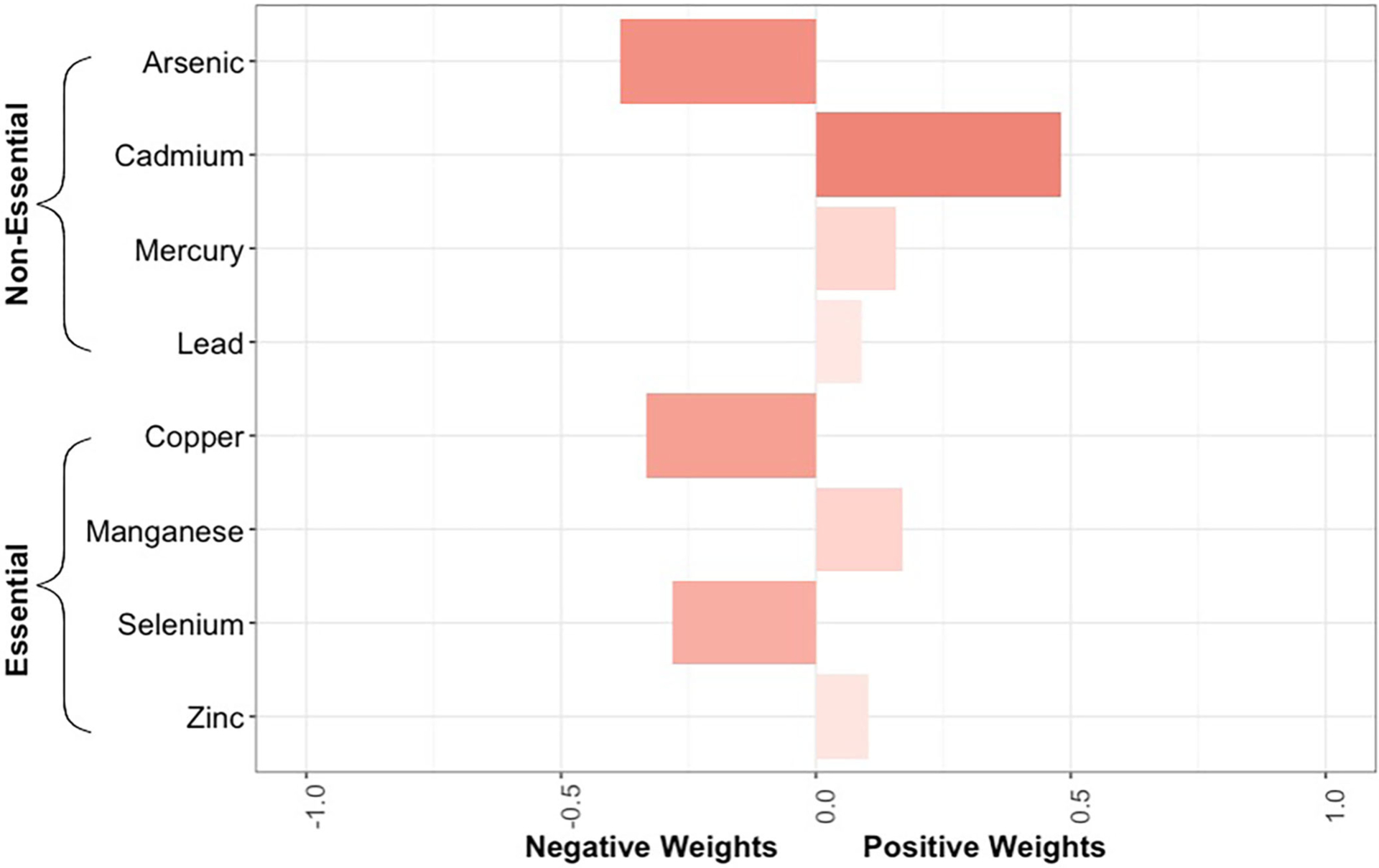
Bar plot showing the weights assigned to each trace element composing the overall mixture as estimated by the quantile g-computation model for neonatal bacterial sepsis in the first 28 days of life after adjustment for geographic region, maternal race/ethnicity, age, educational attainment, Medicaid coverage, pre-pregnancy BMI, and smoking status during pregnancy. The size of the bar corresponds to the proportional contribution of a given trace element to the association with sepsis in a particular direction (negative or positive). Because the size of the bars should only be compared relative to others in the same direction, the shading reflects the absolute contribution of the individual trace element, regardless of directionality.

The quantile g-computation model containing all 8 trace elements estimated an adjusted hazard ratio of 0.92 (95% CI: 0.68–1.25, Figure 3). Of the elements assigned positive weights, cadmium had the largest contribution (Figure 4). Of the elements assigned negative weights, arsenic, copper, and selenium, had the largest contributions. The remaining trace elements were weighted close to zero, indicating negligible associations with incident sepsis. Separating the trace elements into two classes revealed opposing directions of associations: the mixture of non-essential trace elements was associated with an 19% (HR: 1.19, 95% CI: 0.92–1.55) higher risk of sepsis whereas the mixture of essential trace elements was associated with a 23% (HR: 0.77, 95% CI: 0.57–1.06) lower risk (Figure 3).

In quantile g-computation models of late-onset sepsis, the hazard ratios for mixtures of all trace elements and the essential trace elements were similar to estimates for any sepsis, but the hazard ratio for the mixture of non-essential elements was attenuated toward the null (Supplementary Table 5).

In models that were additionally adjusted for hypothesized intermediates (i.e., gestational age, birth weight, chorioamnionitis, infant sex, pPROM, and infections during pregnancy), hazard ratios for the three sets of trace element mixtures with incident sepsis were comparable to those observed in the primary analyses (Supplementary Table 6).

Finally, mixture models stratified by infant sex did not suggest any effect measure modification with *p*-values >0.20 and similar hazard ratios observed among males and females (Supplementary Table 7).

## Discussion

Neonatal bacterial sepsis is a leading cause of infant morbidity and mortality, resulting in a significant healthcare burden (1, 41). Survivors often face neurological sequelae such as cerebral palsy and impaired motor function that have a persistent impact on quality of life (42). Although research suggests that the contribution of environmental factors to sepsis is just as strong as genetics (43), there have been no epidemiologic studies to date on the role of environmental chemical exposures. In this prospective cohort of infants born extremely preterm, we found suggestive evidence that higher levels of prenatal exposure to cadmium were associated with an increased risk of developing bacterial sepsis during the first 28 days of life. However, the results from mixture analyses indicated that joint prenatal exposures to arsenic, copper, and selenium may have a mitigating effect.

Cadmium is found in tobacco smoke and is a contaminant of certain foods (e.g., cereals, bread, and leafy vegetables) (44). Although smoking during pregnancy was associated with notably higher levels of cadmium in umbilical cord tissue, the low prevalence in ELGAN (9.3%) makes it likely that diet was the predominant source of cadmium exposure for this population (45). During pregnancy, cadmium is partially sequestered in the placenta but does cross the placental barrier to reach the developing fetus (46). We found that nearly all umbilical cord tissue samples (98.1%) had detectable levels of cadmium and the median concentration was 1.33 ng/g, a value in line with other studies examining trace elements in cord tissue (47, 48). Prenatal exposure to cadmium has been tied to immunosuppressive endpoints in both animal and epidemiologic studies, including lower levels of T helper memory and natural killer cells (17, 49), potentially increasing susceptibility to infections. There is also evidence to suggest the immunosuppressive effects of prenatal cadmium exposure differ by fetal sex. In mice exposed to environmentally-relevant levels of cadmium *in utero* and later immunized with the streptococcal vaccine, females exhibited a greater reduction in vaccine-induced antibody production and in the number of antibody secreting cells as compared to males (49). In our study, stratified models revealed stronger associations between prenatal cadmium exposure and incident sepsis in female infants, highlighting the potential for sex-based differences in the immunotoxic effects related to prenatal cadmium exposure.

While our results suggested greater prenatal exposure to cadmium increases the risk of neonatal bacterial sepsis, copper, selenium, and arsenic seemed to do the opposite. Although we anticipated that copper and selenium, as essential nutrients, would confer protection, the results for arsenic were contrary to our hypothesis. Arsenic is a non-essential metalloid and prenatal exposures have previously been linked to immunosuppression and inflammation (50, 51). However, it is possible that arsenic exposure could reduce the risk of sepsis, as it was historically used as a treatment for syphilis due to its antimicrobial properties (52). In 1909, Paul Ehrlich and Sahachiro Hata discovered that the organic arsenic compound arsphenamine (later sold under the brand name Salvarsan) was remarkably effective at killing the parasitic bacterium *Treponema pallidum* without overtly harming the host (53). One explanation for our findings is that the infants with the highest cord tissue arsenic concentrations had predominantly been exposed to *organic* arsenic compounds (e.g., arsenobetaine, arsenosugars, arsenolipids), which are frequently found in seafood and are believed to be far less toxic than inorganic forms (54). Since only total arsenic content was measured for this study, we were unable to consider exposure to different arsenic species. It is also plausible that the inverse association observed for arsenic is an artifact of uncontrolled negative confounding by seafood intake (55). In addition to organic arsenicals, seafood, including fish, shellfish, and seaweed, contains nutrients like omega-3 fatty acids that have been shown in an *in vitro* study to induce anti-inflammatory effects relevant for the prevention of neonatal sepsis (56). The ELGAN cohort did not assess prenatal dietary intakes so we were unable to examine seafood consumption during pregnancy as a source of arsenic and/or mercury exposure. However, the strongest element-by-element correlation we observed was between arsenic and mercury. Methylmercury, in particular, is a common seafood contaminant and, like arsenic, requires more complex sample preparation and speciation analysis techniques to determine specific biomarker concentrations (57). Given the very small sample masses of umbilical cord tissue available, speciation analysis was not feasible for this study. Future cohort studies examining environmental trace element exposures should consider collecting prospective dietary data and sufficient biological samples for performing speciation analyses to better disentangle beneficial and harmful effects.

The apparent protective associations observed for copper and selenium in this study are noteworthy and raise the possibility of dietary interventions as preventive or therapeutic strategies. Similar to the issue of seafood consumption during pregnancy, the findings for copper and selenium may be an artifact of uncontrolled confounding by prenatal supplement use, for which detailed information was not collected in ELGAN. Still, experimental data and the biological role of nutritionally essential trace elements provide a rationale for further investigation in the context of neonatal bacterial sepsis prevention (58–60). In a randomized trial of very low birth weight and preterm infants (<1500 g and <32 weeks’, *n* = 90), supplementation of 10 μg selenium per day for 28 days significantly reduced the risk of culture-proven sepsis by 13.3% (60). Both copper and selenium are cofactors for antioxidant enzymes, which suppress the formation of free radicals that, if left unchecked, can lead to cellular and tissue damage (61). Specifically, glutathione peroxidase depends on selenium (62), whereas superoxide dismutase, in forms SOD1 and SOD3, requires copper (in addition to zinc) (63). In the United States, it is estimated that <10% of pregnant women have inadequate copper and selenium intakes, but virtually no one exceeds the respective tolerable upper intake levels (64), indicating there may be an opportunity to increase amounts present in prenatal dietary supplements. Another potential avenue could be including selenium in parenteral nutrition formulations used by preterm infants. In spite of a recommendation by the American Society for Parenteral and Enteral Nutrition, selenium is not a component in the only multi-trace element product currently marketed in North America for neonates (Multitrace-4 Neonatal by American Regent) (65). Investigating supplementation, either during pregnancy or after delivery, for the prevention of neonatal sepsis is a key area for future research (66), and could be expanded to examine efficacy in sub-groups highly exposed to environmental contaminants.

Although there is some evidence for adverse immunological effects of lead (20), mercury (18), manganese (67), and zinc at certain levels (24), associations for these trace elements appeared null in this analysis. This could be due to the particular exposure ranges represented in ELGAN. For instance, compared to umbilical cord tissue concentrations reported in the literature (47, 48), we found lower concentrations of lead and higher concentrations of zinc, suggesting low levels of exposure to lead pollution and adequate zinc nutriture within the ELGAN cohort. Another important consideration is the relatively small sample size, which may have hampered our ability to detect modest associations and left us underpowered to conduct some proposed analyses. Notably, we were unable to investigate early-onset cases of sepsis independently. In models restricted to late-onset cases, the association for the non-essential trace elements mixture was near null. While this finding may indirectly support *in utero* exposures to non-essential trace elements as a more salient risk factor for early as opposed to late-onset sepsis, validation in a larger cohort is needed. Alternatively, we acknowledge that although many non-essential trace elements show evidence for immunotoxicity, it may be that neonatal bacterial sepsis is not a particularly sensitive outcome to exposures in the context of extremely preterm infants for whom other factors (e.g., *in utero* infection status, immune system immaturity, and the need for invasive medical procedures) likely play an outsized role in determining risk.

Despite limitations of this study, notably its small sample size, lack of important dietary information, and inability to perform trace element speciation, it is the first to investigate environmental chemical exposures as a risk factor for neonatal bacterial sepsis. Beyond novelty, there are notable analytic strengths, including the prospective study design, classification of sepsis status through blood culture, and assessment of multiple trace element exposures through biomarkers. Sepsis status was defined by the presence of bacteria in the (usually sterile) bloodstream, indicating bacteremia. At first glance, this definition may seem incomplete because it does not include measures of systemic inflammation. However, markers like C-reactive protein (CRP) and white blood cell counts are generally uninformative in neonates (68). By contrast, blood culture is considered the “gold standard” for diagnosing bacterial sepsis in neonates and was performed serially in ELGAN, improving the internal validity of this study (68). In addition, the use of umbilical cord tissue as a biospecimen to measure prenatal exposure to metallic and metalloid trace elements is a unique advantage. Although most studies rely on maternal samples such as blood or urine collected during pregnancy, maternal biomarkers may not reflect fetal exposures due to toxicokinetics. At present, the literature on trace element content within umbilical cord tissue is sparse and limited data suggest that, compared to umbilical cord blood, tissue is acceptable for assessing *in utero* exposures to mercury and selenium, whereas it may be the inferior matrix for cadmium, lead, copper, and zinc, and there are no data on arsenic or manganese either way (48). In addition, there is the possibility that the location of the tissue sampled affects the amount of trace element present; given that the tissue assessed in this study was obtained from the portion of umbilical cord closest to the abdominal wall, we believe our study appropriately characterized prenatal trace element exposures of mercury and selenium but acknowledge that instead measuring concentrations of the other trace elements in cord blood would have likely enhanced study validity. An additional strength of this study was the availability of rich covariate information, including sociodemographic factors and pregnancy complications that are known risk factors for neonatal sepsis. We leveraged these data to control for potential confounders in primary analyses and hypothesized intermediates in supplemental analyses. The results from both were nearly identical, indicating that, under certain assumptions (69), prenatal metal and metalloid exposures likely influence the risk of neonatal bacterial sepsis directly, rather than through pregnancy complications or infant characteristics.

In summary, we identified a positive association for prenatal cadmium exposure with incident bacterial sepsis in a cohort of extremely preterm infants, which was consistent when cadmium was examined in isolation and within the context of joint exposures to other metals and metalloids. This association was more pronounced among female infants, suggesting cadmium-associated immunotoxicity may differ by sex. It also did not appear to be mediated through pregnancy complications or infant characteristics, indicating cadmium may directly affect the developing immune system. Interestingly, higher levels of *in utero* exposures to arsenic, copper, and selenium seemed to mitigate the risk associated with high cadmium exposures. Future studies are needed to understand whether exposure to specific arsenic species are contributing to this unexpected finding, whether the results could be explained by negative confounding by seafood consumption during pregnancy, and whether copper and selenium supplementation are viable strategies for reducing rates of bacterial sepsis in high-risk infants.

## Supplementary Material

Supplementary Material

## Figures and Tables

**TABLE 1:** Median umbilical cord tissue trace element concentrations, overall and stratified by participant characteristics in the ELGAN study (*n* = 269).

		Non-essential trace elements (ng/g)				Essential trace elements (μg/g)			
	*n* (%)	Arsenic	Cadmium	Mercury	Lead	Copper	Manganese	Selenium	Zinc
**Overall**	269 (100.0)	4.72 (3.32, 7.61)	1.33 (0.76, 3.11)	7.74 (3.66, 17.65)	16.60 (9.30, 31.60)	3.48 (2.97, 4.32)	0.34 (0.30, 0.42)	0.86 (0.77, 0.97)	60.30 (52.23, 71.70)
**Maternal age**									
≤25 years	71 (26.4)	3.80 (2.46, 5.19)	0.99 (0.68, 2.47)	5.62 (2.29, 10.84)	12.60 (8.15, 25.05)	3.26 (2.81, 4.11)	0.34 (0.30, 0.42)	0.83 (0.74, 0.96)	56.30 (46.65, 63.10)
>25–≤30 years	74 (27.5)	5.18 (3.32, 7.14)	1.28 (0.75, 2.41)	6.00 (3.14, 13.68)	13.25 (8.77, 21.92)	3.62 (3.08, 4.39)	0.35 (0.28, 0.42)	0.86 (0.81, 0.98)	61.90 (53.83, 74.07)
>30–≤35 years	62 (23.0)	4.75 (3.39, 9.22)	1.35 (0.77, 3.01)	10.75 (5.27, 17.84)	19.30 (11.20, 31.90)	3.79 (3.13, 4.36)	0.35 (0.30, 0.42)	0.86 (0.77, 0.95)	61.30 (54.27, 72.80)
>35 years	62 (23.0)	6.36 (4.30, 9.80)	1.79 (1.03, 3.73)	15.54 (6.14, 32.62)	21.85 (13.65, 43.50)	3.40 (3.01, 4.58)	0.33 (0.30, 0.42)	0.87 (0.80, 0.96)	61.85 (54.60, 74.93)
**Maternal race/ethnicity**									
Hispanic	20 (7.4)	6.00 (4.38, 8.79)	1.21 (0.82, 2.32)	11.38 (4.58, 18.26)	21.70 (12.60, 34.29)	3.62 (3.35, 4.94)	0.32 (0.28, 0.37)	0.81 (0.73, 0.90)	59.13 (53.20, 66.77)
Non-Hispanic black	73 (27.1)	3.80 (2.50, 6.75)	0.88 (0.58, 2.21)	6.70 (4.34, 13.69)	11.30 (7.40, 19.50)	3.50 (2.87, 4.47)	0.34 (0.28, 0.43)	0.91 (0.77, 0.99)	55.30 (47.60, 62.99)
Non-Hispanic other	10 (3.7)	4.14 (3.32, 5.47)	1.55 (1.02, 3.66)	11.06 (3.48, 14.79)	16.00 (12.62, 26.75)	3.23 (3.02, 3.67)	0.44 (0.39, 0.52)	0.80 (0.71, 0.86)	60.05 (47.60, 69.70)
Non-Hispanic white	166 (61.7)	4.97 (3.62, 8.04)	1.54 (0.86, 3.75)	8.29 (3.40, 18.95)	18.65 (10.12, 35.10)	3.47 (2.97, 4.24)	0.34 (0.30, 0.41)	0.86 (0.78, 0.96)	63.05 (54.94, 77.77)
**Maternal educational attainment**									
Less than high school	34 (12.6)	3.93 (2.45, 5.45)	1.65 (0.79, 3.34)	6.17 (2.99, 14.37)	14.60 (11.93, 32.04)	3.42 (3.03, 4.18)	0.37 (0.30, 0.40)	0.84 (0.75, 0.92)	52.90 (42.42, 63.70)
High school diploma	124 (46.1)	4.37 (2.81, 6.51)	1.19 (0.76, 2.73)	6.58 (3.34, 12.92)	15.00 (8.17, 27.75)	3.45 (2.89, 4.23)	0.33 (0.30, 0.42)	0.85 (0.76, 0.98)	57.80 (48.90, 66.72)
College	111 (41.3)	5.70 (4.01, 9.85)	1.36 (0.75, 3.27)	11.19 (5.31, 26.43)	19.40 (10.90, 33.25)	3.58 (3.00, 4.49)	0.35 (0.30, 0.41)	0.88 (0.80, 0.96)	64.70 (56.25, 75.90)
**Maternal medicaid coverage**									
No	183 (68.0)	5.50 (3.64, 8.93)	1.35 (0.78, 3.00)	10.41 (4.94, 21.55)	18.20 (10.30, 33.99)	3.54 (2.98, 4.24)	0.35 (0.30, 0.42)	0.87 (0.79, 0.97)	62.80 (55.09, 75.50)
Yes	86 (32.0)	3.99 (2.47, 5.40)	1.19 (0.72, 3.42)	5.57 (2.62, 10.46)	12.60 (8.12, 22.28)	3.42 (2.94, 4.55)	0.33 (0.29, 0.42)	0.82 (0.73, 0.94)	54.80 (46.38, 62.53)
**Maternal smoking during pregnancy**									
No	244 (90.7)	4.71 (3.32, 7.89)	1.27 (0.75, 2.86)	8.50 (3.83, 18.04)	16.60 (9.17, 30.85)	3.54 (2.99, 4.46)	0.34 (0.30, 0.42)	0.86 (0.79, 0.97)	61.10 (52.68, 73.32)
Yes	25 (9.3)	4.74 (3.38, 5.50)	2.45 (1.28, 14.89)	5.52 (2.78, 10.86)	14.70 (11.20, 35.90)	3.28 (2.89, 3.87)	0.37 (0.29, 0.40)	0.80 (0.73, 0.96)	54.20 (51.00, 60.10)
**Maternal pre-pregnancy body mass index**									
Normal	167 (62.1)	4.69 (3.42, 7.52)	1.34 (0.78, 2.91)	7.97 (3.33, 19.36)	17.20 (9.65, 32.70)	3.41 (2.88, 4.13)	0.34 (0.30, 0.41)	0.87 (0.78, 0.97)	61.60 (53.25, 73.35)
Obese	58 (21.6)	4.44 (2.80, 6.46)	1.15 (0.69, 3.49)	6.63 (4.06, 11.96)	16.20 (10.08, 29.80)	3.88 (3.21, 4.74)	0.33 (0.28, 0.41)	0.83 (0.73, 0.91)	55.70 (49.00, 62.10)
Overweight	44 (16.4)	5.78 (3.56, 9.81)	1.42 (0.68, 3.39)	10.87 (4.15, 19.56)	11.90 (8.97, 21.33)	3.46 (3.12, 4.23)	0.35 (0.30, 0.46)	0.86 (0.80, 0.97)	63.70 (54.83, 72.83)
**Geographic region**									
Midwest	68 (25.3)	4.65 (3.26, 5.82)	1.60 (0.72, 4.38)	4.20 (2.22, 9.57)	10.72 (6.68, 17.45)	3.59 (2.91, 4.36)	0.32 (0.29, 0.41)	0.93 (0.86, 1.00)	65.30 (56.22, 79.35)
New England	121 (45.0)	5.96 (4.20, 9.90)	1.55 (0.85, 3.31)	15.05 (8.13, 32.04)	23.90 (17.00, 43.40)	3.54 (3.07, 4.42)	0.36 (0.31, 0.43)	0.87 (0.80, 0.96)	63.60 (56.59, 80.30)
North Carolina	80 (29.7)	3.32 (2.32, 5.20)	1.03 (0.70, 1.96)	6.00 (3.26, 8.73)	11.15 (7.18, 19.27)	3.30 (2.89, 4.01)	0.32 (0.28, 0.42)	0.77 (0.70, 0.85)	50.45 (44.27, 58.02)
**Infant sex**									
Male	149 (55.4)	4.73 (3.32, 8.11)	1.35 (0.81, 3.31)	8.36 (4.02, 20.23)	18.20 (10.83, 34.00)	3.59 (3.02, 4.53)	0.34 (0.30, 0.41)	0.85 (0.79, 0.96)	61.50 (53.95, 73.30)
Female	120 (44.6)	4.70 (3.32, 6.95)	1.25 (0.73, 2.49)	7.32 (3.43, 15.06)	14.40 (8.82, 25.83)	3.39 (2.88, 4.09)	0.34 (0.30, 0.42)	0.86 (0.77, 0.97)	57.94 (50.94, 69.90)
**Neonatal bacterial Sepsis**									
No	188 (69.9)	5.05 (3.42, 8.64)	1.22 (0.74, 2.59)	8.56 (3.74, 20.22)	16.80 (9.97, 31.38)	3.54 (3.02, 4.46)	0.34 (0.30, 0.42)	0.86 (0.79, 0.97)	59.68 (52.55, 70.33)
Yes	81 (30.1)	4.51 (3.07, 5.70)	1.84 (0.86, 3.98)	6.19 (3.35, 13.40)	16.30 (8.70, 32.90)	3.39 (2.86, 3.99)	0.34 (0.29, 0.43)	0.84 (0.75, 0.93)	60.90 (51.70, 78.90)
Early-onset	19 (7.1)	5.00 (3.71, 7.13)	2.15 (1.41, 4.80)	12.63 (5.34, 21.13)	16.30 (9.45, 31.40)	3.83 (2.69, 4.19)	0.34 (0.29, 0.45)	0.87 (0.82, 0.92)	57.00 (51.90, 76.15)
Late-onset	62 (23.0)	4.14 (2.84, 5.69)	1.77 (0.85, 3.93)	5.46 (2.84, 11.32)	16.17 (7.92, 32.94)	3.33 (2.89, 3.85)	0.34 (0.29, 0.41)	0.84 (0.74, 0.94)	61.04 (51.75, 78.90)

Values inside the parenthesis represent the 25th and 75th percentiles.

## Data Availability

The data that support the findings of this study are available upon reasonable request from the corresponding author, RCF. The data are not available publicly due to their containing information that could compromise the privacy of the ELGAN study participants.

## References

[1] Fleischmann C, Reichert F, Cassini A, Horner R, Harder T, Markwart R, Global incidence and mortality of neonatal sepsis: a systematic review and meta-analysis. Arch Dis Child. (2021) 106:745–52. doi: 10.1136/archdischild-2020-320217 PMC8311109 33483376

[2] Wortham JM, Hansen NI, Schrag SJ, Hale E, Van Meurs K, Sanchez PJ, Chorioamnionitis and culture-confirmed, early-onset neonatal infections. Pediatrics. (2016) 137. doi: 10.1542/peds.2015-2323 PMC4702021 26719293

[3] Stoll BJ, Puopolo KM, Hansen NI, Sanchez PJ, Bell EF, Carlo WA, Human development neonatal research, early-onset neonatal sepsis 2015 to 2017, the rise of Escherichia coli, and the Need for Novel Prevention Strategies. JAMA Pediatr. (2020) 174:e200593. doi: 10.1001/jamapediatrics.2020.0593 32364598 PMC7199167

[4] Tchounwou PB, Yedjou CG, Patlolla AK, Sutton DJ. Heavy metal toxicity and the environment. Exp Suppl. (2012) 101:133–64. doi: 10.1007/978-3-7643-8340-4_6 22945569 PMC4144270

[5] Dangleben NL, Skibola CF, Smith MT. Arsenic immunotoxicity: a review. Environ Health. (2013) 12:73. doi: 10.1186/1476-069X-12-73 24004508 PMC3848751

[6] Wang Z, Sun Y, Yao W, Ba Q, Wang H. Effects of cadmium exposure on the immune system and immunoregulation. Front Immunol. (2021) 12:695484. doi: 10.3389/fimmu.2021.695484 34354707 PMC8330548

[7] Dietert RR, Lee JE, Hussain I, Piepenbrink M. Developmental immunotoxicology of lead. Toxicol Appl Pharmacol. (2004) 198:86–94. doi: 10.1016/j.taap.2003.08.020 15236947

[8] Tchounwou PB, Ayensu WK, Ninashvili N, Sutton D. Environmental exposure to mercury and its toxicopathologic implications for public health. Environ Toxicol. (2003) 18:149–75. doi: 10.1002/tox.10116 12740802

[9] Percival SS. Copper and immunity. Am J Clin Nutr. (1998) 67:1064S–8S. doi: 10.1093/ajcn/67.5.1064S 9587153

[10] Kehl-Fie TE, Skaar EP. Nutritional immunity beyond iron: a role for manganese and zinc. Curr Opin Chem Biol. (2010) 14:218–24. doi: 10.1016/j.cbpa.2009.11.008 20015678 PMC2847644

[11] Arthur JR, McKenzie RC, Beckett GJ. Selenium in the immune system. J Nutr. (2003) 133 (5 Suppl 1): 1457S–9S. doi: 10.1093/jn/133.5.1457S 12730442

[12] Beck MA, Nelson HK, Shi Q, Van Dael P, Schiffrin EJ, Blum S, Selenium deficiency increases the pathology of an influenza virus infection. FASEB J. (2001) 15:1481–3. doi: 10.1096/fj.00-0721fje 11387264

[13] Haynes DC, Gershwin ME, Golub MS, Cheung AT, Hurley LS, Hendrickx AG, Studies of marginal zinc deprivation in rhesus monkeys: VI. Influence on the immunohematology of infants in the first year. Am J Clin Nutr. (1985) 42:252–62. doi: 10.1093/ajcn/42.2.252 4025197

[14] Juttukonda LJ, Berends ETM, Zackular JP, Moore JL, Stier MT, Zhang Y, Dietary manganese promotes staphylococcal infection of the heart. Cell Host Microbe. (2017). 22:531–42.e8. doi: 10.1016/j.chom.2017.08.009 28943329 PMC5638708

[15] Pocino M, Baute L, Malave I. Influence of the oral administration of excess copper on the immune response. Fundam Appl Toxicol. (1991) 16:249–56. doi: 10.1016/0272-0590(91)90109-H 2055356

[16] Soto-Pena GA, Luna AL, Acosta-Saavedra L, Conde P, Lopez-Carrillo L, Cebrian ME, Assessment of lymphocyte subpopulations and cytokine secretion in children exposed to arsenic. FASEB J. (2006) 20:779–81. doi: 10.1096/fj.05-4860fje 16461332

[17] Nygaard UC, Li Z, Palys T, Jackson B, Subbiah M, Malipatlolla M, Cord blood T cell subpopulations and associations with maternal cadmium and arsenic exposures. PLoS ONE. (2017) 12:e0179606. doi: 10.1371/journal.pone.0179606 28662050 PMC5491028

[18] Oulhote Y, Shamim Z, Kielsen K, Weihe P, Grandjean P, Ryder LP, Children’s white blood cell counts in relation to developmental exposures to methylmercury and persistent organic pollutants. Reprod Toxicol. (2017) 68:207–14. doi: 10.1016/j.reprotox.2016.08.001 27497749 PMC5292093

[19] Chaudhry H, Zhou J, Zhong Y, Ali MM, McGuire F, Nagarkatti PS, Role of cytokines as a double-edged sword in sepsis. In Vivo. (2013) 27:669–84. 24292568 PMC4378830

[20] Wang M, Xia W, Zeng Q, Zhang W, Qian X, Bao S, Associations between prenatal and postnatal lead exposure and preschool children humoral and cellular immune responses. Ecotoxicol Environ Saf. (2021) 207:111536. doi: 10.1016/j.ecoenv.2020.111536 33254398

[21] Bulka CM, Bommarito PA, Fry RC. Predictors of toxic metal exposures among US women of reproductive age. J Expo Sci Environ Epidemiol. (2019) 29:597–612. doi: 10.1038/s41370-019-0152-3 31235790 PMC6709576

[22] Bridges CC, Zalups RK. Molecular and ionic mimicry and the transport of toxic metals. Toxicol Appl Pharmacol. (2005) 204:274–308. doi: 10.1016/j.taap.2004.09.007 15845419 PMC2409291

[23] Breton CV, Farzan SF. Invited perspective: metal mixtures and child health: the complex interplay of essential and toxic elements. Environ Health Perspect. (2021) 129:61301. doi: 10.1289/EHP9629 34160248 PMC8312474

[24] Liu MJ, Bao S, Galvez-Peralta M, Pyle CJ, Rudawsky AC, Pavlovicz RE, ZIP8 regulates host defense through zinc-mediated inhibition of NF-kappaB. Cell Rep. (2013) 3:386–400. doi: 10.1016/j.celrep.2013.01.009 23403290 PMC3615478

[25] Singh M, Alsaleem M, Gray CP. Neonatal sepsis. Treasure Island (FL): StatPearls (2022). 30285373

[26] O’Shea TM, Allred EN, Dammann O, Hirtz D, Kuban KC, Paneth N, The ELGAN study of the brain and related disorders in extremely low gestational age newborns. Early Hum Dev. (2009) 85:719–25. doi: 10.1016/j.earlhumdev.2009.08.060 19765918 PMC2801579

[27] Patel S, Dammann O, Martin CR, Allred EN, Leviton A, Investigators ES, Presumed and definite bacteremia in extremely low gestational age newborns. Acta Paediatr. (2011) 100:36–41. doi: 10.1111/j.1651-2227.2010.01963.x 20712830 PMC3006000

[28] Stoll BJ, Hansen NI, Sanchez PJ, Faix RG, Poindexter BB, Van Meurs KP, Early onset neonatal sepsis: the burden of group B Streptococcal and *E. coli* disease continues. Pediatrics. (2011) 127:817–26. doi: 10.1542/peds.2010-2217 21518717 PMC3081183

[29] Dong Y, Speer CP. Late-onset neonatal sepsis: recent developments. Arch Dis Child Fetal Neonatal Ed. (2015) 100:F257–63. doi: 10.1136/archdischild-2014-306213 25425653 PMC4413803

[30] Textor J, van der Zander B, Gilthorpe MS, Liskiewicz M, Ellison GT. Robust causal inference using directed acyclic graphs: the R package ‘dagitty’. Int J Epidemiol. (2016) 45:1887–94. doi: 10.1093/ije/dyw341 28089956

[31] R Core Team. R: A Language and Environment for Statistical Computing. R Foundation for Statistical Computing, Vienna, Austria. (2020). Available Online at: https://www.R-project.org/

[32] Richardson DB. Discrete time hazards models for occupational and environmental cohort analyses. Occup Environ Med. (2010) 67:67–71. doi: 10.1136/oem.2008.044834 20029026

[33] Singer JD, Willett JB. It’s about time: using discrete-time survival analysis to study duration and the timing of events. J Educ Stat. (1993) 18:155–95. doi: 10.3102/10769986018002155

[34] Cameron AC, Gelbach JB. Bootstrap-based improvements for inference with clustered errors. Rev Econ Stat. (2008) 90:414–27. doi: 10.1162/rest.90.3.414

[35] Greenland S Avoiding power loss associated with categorization and ordinal scores in dose-response and trend analysis. Epidemiology. (1995) 6:450–4. doi: 10.1097/00001648-199507000-00025 7548361

[36] Keil AP, Buckley JP, O’Brien KM, Ferguson KK, Zhao S, White AJ, A quantile-based g-computation approach to addressing the effects of exposure mixtures. Environ Health Perspect. (2020) 128:47004. doi: 10.1289/EHP5838 32255670 PMC7228100

[37] Rahman ML, Oken E, Hivert MF, Rifas-Shiman S, Lin PD, Colicino E, Early pregnancy exposure to metal mixture and birth outcomes—a prospective study in Project Viva. Environ Int. (2021) 156:106714. doi: 10.1016/j.envint.2021.106714 34147999 PMC8842844

[38] Venkatesh KK, Leviton A, Hecht JL, Joseph RM, Douglass LM, Frazier JA, Histologic chorioamnionitis and risk of neurodevelopmental impairment at age 10 years among extremely preterm infants born before 28 weeks of gestation. Am J Obstet Gynecol. (2020) 223:745.e1–e10. doi: 10.1016/j.ajog.2020.05.001 32387324 PMC7609587

[39] Hanson ML, Holaskova I, Elliott M, Brundage KM, Schafer R, Barnett JB, Prenatal cadmium exposure alters postnatal immune cell development and function. Toxicol Appl Pharmacol. (2012) 261:196–203. doi: 10.1016/j.taap.2012.04.002 22521604 PMC3358511

[40] Buckley JP, Doherty BT, Keil AP, Engel SM. Statistical approaches for estimating sex-specific effects in endocrine disruptors research. Environ Health Perspect. (2017) 125:067013. doi: 10.1289/EHP334 28665274 PMC5743445

[41] Ely DM, Driscoll AK, Matthews TJ. Infant mortality by age at death in the United States, 2016. NCHS Data Brief. (2018) 326: 1–8. 30475688

[42] Dahlem P, Biggar P. Follow-up of newborns, infants, and children with sepsis. J Child Sci. (2017) 07:e38–41. doi: 10.1055/s-0037-1603893

[43] Bizzarro MJ, Jiang Y, Hussain N, Gruen JR, Bhandari V, Zhang H, The impact of environmental and genetic factors on neonatal late-onset sepsis. J Pediatr. (2011) 158, 234–8.e1. doi: 10.1016/j.jpeds.2010.07.060 20850766 PMC3008342

[44] Kim K, Melough MM, Vance TM, Noh H, Koo SI, Chun OK, Dietary cadmium intake and sources in the US. Nutrients. (2018) 11: nu11010002. doi: 10.3390/nu11010002 PMC6356330 30577418

[45] Faroon O, Ashizawa A, Wright S, Tucker P, Jenkins K, Ingerman L, Toxicological Profile for Cadmium. (2012). Atlanta (GA): CDC. 24049863

[46] Punshon T, Li Z, Marsit CJ, Jackson BP, Baker ER, Karagas MR, Placental metal concentrations in relation to maternal and infant toenails in a U.S. cohort. Environ Sci Technol. (2016) 50:1587–94. doi: 10.1021/acs.est.5b05316 26727403 PMC4873612

[47] Ni W, Yang W, Yu J, Li Z, Jin L, Liu J, Umbilical cord concentrations of selected heavy metals and risk for orofacial clefts. Environ Sci Technol. (2018) 52:10787–95. doi: 10.1021/acs.est.8b02404 30134103

[48] Sakamoto M, Yasutake A, Domingo JL, Chan HM, Kubota M, Murata K, Relationships between trace element concentrations in chorionic tissue of placenta and umbilical cord tissue: potential use as indicators for prenatal exposure. Environ Int. (2013) 60:106–11. doi: 10.1016/j.envint.2013.08.007 24028800

[49] Holaskova I, Elliott M, Hanson ML, Schafer R, Barnett JB. Prenatal cadmium exposure produces persistent changes to thymus and spleen cell phenotypic repertoire as well as the acquired immune response. Toxicol Appl Pharmacol. (2012) 265:181–9. doi: 10.1016/j.taap.2012.10.009 23088857 PMC3508345

[50] Ahmed S, Ahsan KB, Kippler M, Mily A, Wagatsuma Y, Hoque AM, In utero arsenic exposure is associated with impaired thymic function in newborns possibly via oxidative stress and apoptosis. Toxicol Sci. (2012) 129:305–14. doi: 10.1093/toxsci/kfs202 22713597

[51] Fry RC, Navasumrit P, Valiathan C, Svensson JP, Hogan BJ, Luo M, Activation of inflammation/NF-kappaB signaling in infants born to arsenic-exposed mothers. PLoS Genet. (2007) 3:e207. doi: 10.1371/journal.pgen.0030207 18039032 PMC2082467

[52] Thorburn AL. Paul Ehrlich: pioneer of chemotherapy and cure by arsenic (1854–1915). Br J Vener Dis. (1983) 59:404–5. doi: 10.1136/sti.59.6.404 6196079 PMC1046247

[53] Frith J Arsenic—The “Poison of Kings” and The “Saviour of Syphilis”. (2013). Hobart: Australian Military Medical Association Inc., p. 11–7.

[54] Jones MR, Tellez-Plaza M, Vaidya D, Grau M, Francesconi KA, Goessler W, Estimation of inorganic arsenic exposure in populations with frequent seafood intake: evidence from MESA and NHANES. Am J Epidemiol. (2016) 184:590–602. doi: 10.1093/aje/kww097 27702745 PMC5065621

[55] Choi AL, Cordier S, Weihe P, Grandjean P. Negative confounding in the evaluation of toxicity: the case of methylmercury in fish and seafood. Crit Rev Toxicol. (2008) 38:877–93. doi: 10.1080/10408440802273164 19012089 PMC2597522

[56] Espiritu MM, Lin H, Foley E, Tsang V, Rhee E, Perlman J, Omega-3 fatty acids modulate neonatal cytokine response to endotoxin. J Perinat Med. (2016) 44:711–21. doi: 10.1515/jpm-2015-0248 26812855

[57] Suvarapu LN, Baek S-O. Recent studies on the speciation and determination of mercury in different environmental matrices using various analytical techniques. Int J Anal Chem. (2017) 2017:3624015. doi: 10.1155/2017/3624015 29348750 PMC5733771

[58] Tang Z, Wei Z, Wen F, Wu Y. Efficacy of zinc supplementation for neonatal sepsis: a systematic review and meta-analysis. J Matern Fetal Neonatal Med. (2019) 32:1213–8. doi: 10.1080/14767058.2017.1402001 29103346

[59] Kong Z, Wang F, Ji S, Deng X, Xia Z. Selenium supplementation for sepsis: a meta-analysis of randomized controlled trials. Am J Emerg Med. (2013) 31:1170–5. doi: 10.1016/j.ajem.2013.04.020 23791608

[60] Aggarwal R, Gathwala G, Yadav S, Kumar P. Selenium supplementation for prevention of late-onset sepsis in very low birth weight preterm neonates. J Trop Pediatr. (2016) 62:185–93. doi: 10.1093/tropej/fmv096 26867560

[61] Perrone S, Negro S, Tataranno ML, Buonocore G. Oxidative stress and antioxidant strategies in newborns. J Matern Fetal Neonatal Med. (2010) 23 Suppl 3:63–5. doi: 10.3109/14767058.2010.509940 20807155

[62] Institute of Medicine (US) Panel on Dietary Antioxidants and Related Compounds. Dietary Reference Intakes for Vitamin C, Vitamin E, Selenium, and Carotenoids. (2000). Washington (DC): National Academies Press. 25077263

[63] Institute of Medicine (US) Panel on Dietary Antioxidants and Related Compounds. Dietary Reference Intakes for Vitamin A, Vitamin K, Arsenic, Boron, Chromium, Copper, Iodine, Iron, Manganese, Molybdenum, Nickel, Silicon, Vanadium, and Zinc. (2001). Washington (DC): National Academies Press. 25057538

[64] Bailey RL, Pac SG, Fulgoni VL 3rd, Reidy KC, Catalano PM. Estimation of total usual dietary intakes of pregnant women in the United States. JAMA Netw Open. (2019) 2:195967. doi: 10.1001/jamanetworkopen.2019.5967 PMC6593963 31225890

[65] Vanek VW, Borum P, Buchman A, Fessler TA, Howard L, Jeejeebhoy K, ASPEN Position Paper. Nutr. Clin. Pract (2012) 27:440–91. doi: 10.1177/0884533612446706 22730042

[66] Tindell RT. Tipple. Selenium: implications for outcomes in extremely preterm infants. J Perinatol. (2018) 38:197–202. doi: 10.1038/s41372-017-0033-3 29298985 PMC5967885

[67] Chirase NK, Greene LW. Dietary zinc and manganese sources administered from the fetal stage onwards affect immune response of transit stressed and virus infected offspring steer calves. Anim Feed Sci Technol. (2001) 93:217–28. doi: 10.1016/S0377-8401(01)00277-2

[68] Zea-Vera A, Ochoa TJ. Challenges in the diagnosis and management of neonatal sepsis. J Trop Pediatr. (2015) 61:1–13. doi: 10.1093/tropej/fmu079 25604489 PMC4375388

[69] VanderWeele TJ. Mediation analysis: a practitioner’s guide. Annu Rev Public Health. (2016) 37:17–32. doi: 10.1146/annurev-publhealth-032315-021402 26653405

