# Supplementary material for: Prenatal exposure to multiple metallic and metalloid trace elements and the risk of bacterial sepsis in extremely low gestational age newborns: A prospective cohort study
Source: Front Epidemiol. 2022 Sep 7;2:958389. doi: 10.3389/fepid.2022.958389 (PMC9674331; doi:10.3389/fepid.2022.958389)
Supplement: Supplementary file 1 [file Data_Sheet_1.docx]

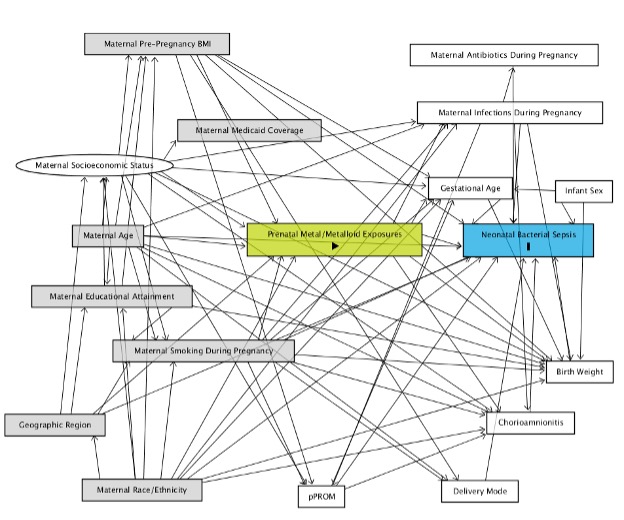
**Supplemental Figure 1.** Directed acyclic graph (DAG) showing hypothesized relationships between prenatal metal/metalloid exposures (green) and neonatal bacterial sepsis (blue).*.* Potential confounders are depicted in gray whereas intermediates and their descendants are depicted in white. Boxes indicate observed variables while the circle around maternal socioeconomic status indicates it was latent (unobserved).

| **Supplemental Table 1. Adjusted associations of umbilical cord tissue trace element concentrations with neonatal bacterial sepsis in the ELGAN study (n=269)** | | | | | |
| --- | --- | --- | --- | --- | --- |
| **Non-Essential Trace Elements** | **HR (95% CI)** | **p_trend_** | **Essential Trace Elements** | **HR (95% CI)** | **p_trend_** |
| **Arsenic (ng/g)** |  | 0.16 | **Copper (μg/g)** |  | 0.17 |
| Q1 (1.26-3.32) | 1.00 (reference) |  | Q1 (1.62-2.97) | 1.00 (reference) |  |
| Q2 (3.33-4.72) | 0.89 (0.41-1.90) |  | Q2 (2.98-3.48) | 0.70 (0.34-1.45) |  |
| Q3 (4.73-7.61) | 0.93 (0.45-1.90) |  | Q3 (3.49-4.32) | 0.60 (0.30-1.22) |  |
| Q4 (7.62-77.70) | 0.55 (0.23-1.30) |  | Q4 (4.33-14.31) | 0.58 (0.27-1.22) |  |
| **Cadmium (ng/g)** |  | 0.18 | **Manganese (μg/g)** |  | 0.68 |
| Q1 (0.05-0.76) | 1.00 (reference) |  | Q1 (0.10-0.30) | 1.00 (reference) |  |
| Q2 (0.77-1.33) | 1.13 (0.51-2.54) |  | Q2 (0.31-0.34) | 0.86 (0.41-1.77) |  |
| Q3 (1.34-3.11) | 1.94 (0.87-4.32) |  | Q3 (0.35-0.42) | 1.00 (0.47-2.10) |  |
| Q4 (3.12-4057.21) | 1.88 (0.90-3.93) |  | Q4 (0.43-5.58) | 1.09 (0.52-2.27) |  |
| **Mercury (ng/g)** |  | 0.59 | **Selenium (μg/g)** |  | 0.16 |
| Q1 (0.56-3.66) | 1.00 (reference) |  | Q1 (0.44-0.77) | 1.00 (reference) |  |
| Q2 (3.66-7.74) | 1.22 (0.62-2.40) |  | Q2 (0.78-0.86) | 0.92 (0.45-1.88) |  |
| Q3 (7.75-17.65) | 1.09 (0.50-2.41) |  | Q3 (0.87-0.97) | 0.82 (0.36-1.90) |  |
| Q4 (17.76-174.11) | 0.88 (0.36-2.17) |  | Q4 (0.98-1.98) | 0.60 (0.28-1.29) |  |
| **Lead (ng/g)** |  | 0.39 | **Zinc (μg/g)** |  | 0.37 |
| Q1 (2.60-9.30) | 1.00 (reference) |  | Q1 (29.87-52.23) | 1.00 (reference) |  |
| Q2 (9.31-16.60) | 0.73 (0.33-1.63) |  | Q2 (52.24-60.30) | 0.58 (0.23-1.47) |  |
| Q3 (16.61-31.60) | 1.01 (0.47-2.16) |  | Q3 (60.31-71.70) | 0.84 (0.37-1.93) |  |
| Q4 (31.61-893.10) | 1.14 (0.56-2.35) |  | Q4 (71.71-487.50) | 1.08 (0.44-2.66) |  |
| Hazard ratios (95% CI) for neonatal bacterial sepsis by umbilical cord tissue trace element concentration quartiles as estimated by separate discrete-time hazard regression models adjusted for geographic region (Midwest, New England, North Carolina), maternal race/ethnicity (Hispanic, non-Hispanic black, non-Hispanic other, non-Hispanic white), age (years), educational attainment (less than high school diploma, high school diploma, college degree), Medicaid coverage (yes or no), pre-pregnancy BMI (kg/m^2^), and smoking status during pregnancy (yes or no). p_trend_ for linear associations with sepsis calculated by modeling the median trace element concentration in each quartile as a continuous variable. | | | | | |

| **Supplemental Table 2. Adjusted associations of umbilical cord tissue trace element concentrations with late-onset neonatal bacterial sepsis in the ELGAN study (n=269)** | | | | | |
| --- | --- | --- | --- | --- | --- |
| **Non-Essential Trace Elements** | **HR (95% CI)** | **p_trend_** | **Essential Trace Elements** | **HR (95% CI)** | **p_trend_** |
| **Arsenic (ng/g)** |  | 0.16 | **Copper (μg/g)** |  | 0.19 |
| Q1 (1.26-3.32) | 1.00 (reference) |  | Q1 (1.62-2.97) | 1.00 (reference) |  |
| Q2 (3.33-4.72) | 0.89 (0.37-2.13) |  | Q2 (2.98-3.48) | 0.90 (0.37-2.22) |  |
| Q3 (4.73-7.61) | 0.95 (0.40-2.29) |  | Q3 (3.49-4.32) | 0.51 (0.20-1.31) |  |
| Q4 (7.62-77.70) | 0.40 (0.12-1.32) |  | Q4 (4.33-14.31) | 0.67 (0.28-1.63) |  |
| **Cadmium (ng/g)** |  | 0.14 | **Manganese (μg/g)** |  | 0.92 |
| Q1 (0.05-0.76) | 1.00 (reference) |  | Q1 (0.10-0.30) | 1.00 (reference) |  |
| Q2 (0.77-1.33) | 1.17 (0.43-3.18) |  | Q2 (0.31-0.34) | 0.93 (0.40-2.18) |  |
| Q3 (1.34-3.11) | 1.30 (0.48-3.52) |  | Q3 (0.35-0.42) | 1.03 (0.43-2.47) |  |
| Q4 (3.12-4057.21) | 1.90 (0.78-4.62) |  | Q4 (0.43-5.58) | 1.02 (0.43-2.40) |  |
| **Mercury (ng/g)** |  | 0.27 | **Selenium (μg/g)** |  | 0.17 |
| Q1 (0.56-3.66) | 1.00 (reference) |  | Q1 (0.44-0.77) | 1.00 (reference) |  |
| Q2 (3.66-7.74) | 1.07 (0.49-2.31) |  | Q2 (0.78-0.86) | 0.88 (0.39-1.97) |  |
| Q3 (7.75-17.65) | 0.78 (0.29-2.13) |  | Q3 (0.87-0.97) | 0.45 (0.15-1.29) |  |
| Q4 (17.76-174.11) | 0.53 (0.17-1.66) |  | Q4 (0.98-1.98) | 0.59 (0.24-1.41) |  |
| **Lead (ng/g)** |  | 0.45 | **Zinc (μg/g)** |  | 0.29 |
| Q1 (2.60-9.30) | 1.00 (reference) |  | Q1 (29.87-52.23) | 1.00 (reference) |  |
| Q2 (9.31-16.60) | 0.63 (0.26-1.55) |  | Q2 (52.24-60.30) | 0.66 (0.24-1.86) |  |
| Q3 (16.61-31.60) | 1.09 (0.44-2.73) |  | Q3 (60.31-71.70) | 1.11 (0.45-2.78) |  |
| Q4 (31.61-893.10) | 1.25 (0.51-3.05) |  | Q4 (71.71-487.50) | 1.37 (0.52-3.59) |  |
| Hazard ratios (95% CI) for neonatal bacterial sepsis by umbilical cord tissue trace element concentration quartiles as estimated by separate discrete-time hazard regression models adjusted for geographic region (Midwest, New England, North Carolina), maternal race/ethnicity (Hispanic, non-Hispanic black, non-Hispanic other, non-Hispanic white), age (years), educational attainment (less than high school diploma, high school diploma, college degree), Medicaid coverage (yes or no), pre-pregnancy BMI (kg/m^2^), and smoking status during pregnancy (yes or no). p_trend_ for linear associations with sepsis calculated by modeling the median trace element concentration in each quartile as a continuous variable. | | | | | |

| **Supplemental Table 3. Intermediate-adjusted associations of umbilical cord tissue trace element concentrations with neonatal bacterial sepsis in the ELGAN study (n=250*)** | | | | | |
| --- | --- | --- | --- | --- | --- |
| **Non-Essential Trace Elements** | **HR (95% CI)** | **p_trend_** | **Essential Trace Elements** | **HR (95% CI)** | **p_trend_** |
| **Arsenic (ng/g)** |  | 0.20 | **Copper (μg/g)** |  | 0.08 |
| Q1 (1.26-3.32) | 1.00 (reference) |  | Q1 (1.62-2.97) | 1.00 (reference) |  |
| Q2 (3.33-4.72) | 0.83 (0.35-1.92) |  | Q2 (2.98-3.48) | 0.59 (0.26-1.32) |  |
| Q3 (4.73-7.61) | 0.86 (0.39-1.89) |  | Q3 (3.49-4.32) | 0.53 (0.24-1.16) |  |
| Q4 (7.62-77.70) | 0.49 (0.18-1.37) |  | Q4 (4.33-14.31) | 0.49 (0.20-1.21) |  |
| **Cadmium (ng/g)** |  | 0.08 | **Manganese (μg/g)** |  | 0.64 |
| Q1 (0.05-0.76) | 1.00 (reference) |  | Q1 (0.10-0.30) | 1.00 (reference) |  |
| Q2 (0.77-1.33) | 1.22 (0.50-2.97) |  | Q2 (0.31-0.34) | 0.65 (0.28-1.51) |  |
| Q3 (1.34-3.11) | 1.97 (0.85-4.59) |  | Q3 (0.35-0.42) | 0.75 (0.31-1.85) |  |
| Q4 (3.12-4057.21) | 1.89 (0.80-4.45) |  | Q4 (0.43-5.58) | 0.79 (0.33-1.85) |  |
| **Mercury (ng/g)** |  | 0.53 | **Selenium (μg/g)** |  | 0.08 |
| Q1 (0.56-3.66) | 1.00 (reference) |  | Q1 (0.44-0.77) | 1.00 (reference) |  |
| Q2 (3.66-7.74) | 1.32 (0.63-2.74) |  | Q2 (0.78-0.86) | 0.89 (0.40-2.01) |  |
| Q3 (7.75-17.65) | 1.11 (0.46-2.70) |  | Q3 (0.87-0.97) | 0.74 (0.28-1.96) |  |
| Q4 (17.76-174.11) | 0.70 (0.25-1.95) |  | Q4 (0.98-1.98) | 0.44 (0.16-1.21) |  |
| **Lead (ng/g)** |  | 0.52 | **Zinc (μg/g)** |  | 0.74 |
| Q1 (2.60-9.30) | 1.00 (reference) |  | Q1 (29.87-52.23) | 1.00 (reference) |  |
| Q2 (9.31-16.60) | 0.71 (0.30-1.68) |  | Q2 (52.24-60.30) | 0.51 (0.18-1.42) |  |
| Q3 (16.61-31.60) | 1.30 (0.56-3.04) |  | Q3 (60.31-71.70) | 0.92 (0.39-2.20) |  |
| Q4 (31.61-893.10) | 1.12 (0.52-2.45) |  | Q4 (71.71-487.50) | 0.89 (0.31-2.59) |  |
| Hazard ratios (95% CI) for neonatal bacterial sepsis by umbilical cord tissue trace element concentration quartiles as estimated by separate discrete-time hazard regression models adjusted for geographic region (Midwest, New England, North Carolina), maternal race/ethnicity (Hispanic, non-Hispanic black, non-Hispanic other, non-Hispanic white), age (years), educational attainment (less than high school diploma, high school diploma, college degree), Medicaid coverage (yes or no), pre-pregnancy BMI (kg/m^2^), smoking status during pregnancy (yes or no), gestational age (weeks), birth weight (grams), chorioamnionitis (yes or no), infections during pregnancy (yes or no), infant sex (female or male), and pPROM (yes or no). p_trend_ for linear associations with sepsis calculated by modeling the median trace element concentration in each quartile as a continuous variable. *19 participants were missing placental histology to confirm a diagnosis of chorioamnionitis. | | | | | |

| **Supplemental Table 4. Adjusted associations of umbilical cord tissue trace element concentrations with neonatal bacterial sepsis by infant sex in the ELGAN study (n=269)** | | | | | | | |
| --- | --- | --- | --- | --- | --- | --- | --- |
|  | **Females**  **(n=120)** | **Males**  **(n=149)** |  |  | **Females**  **(n=120)** | **Males**  **(n=149)** |  |
| **Non-Essential Trace Elements** | **HR (95% CI)** | **HR (95% CI)** | **p_heterogeneity_** | **Essential Trace Elements** | **HR (95% CI)** | **HR (95% CI)** | **p_heterogeneity_** |
| **Arsenic (ng/g)** |  |  |  | **Copper (μg/g)** |  |  |  |
| Q1 (1.26-3.32) | 1.00 (reference) | 1.00 (reference) |  | Q1 (1.62-2.97) | 1.00 (reference) | 1.00 (reference) |  |
| Q2 (3.33-4.72) | 2.22 (0.37-13.52) | 0.36 (0.10-1.37) | 0.41 | Q2 (2.98-3.48) | 1.21 (0.29-4.98) | 0.37 (0.10-1.36) | 0.51 |
| Q3 (4.73-7.61) | 1.51 (0.25-9.17) | 0.56 (0.14-2.19) | 0.55 | Q3 (3.49-4.32) | 0.66 (0.13-3.29) | 0.63 (0.20-2.00) | 0.97 |
| Q4 (7.62-77.70) | 0.70 (0.04-13.30) | 0.46 (0.12-1.79) | 0.77 | Q4 (4.33-14.31) | 0.99 (0.22-4.34) | 0.37 (0.11-1.25) | 0.56 |
| **Cadmium (ng/g)** |  |  |  | **Manganese (μg/g)** |  |  |  |
| Q1 (0.05-0.76) | 1.00 (reference) | 1.00 (reference) |  | Q1 (0.10-0.30) | 1.00 (reference) | 1.00 (reference) |  |
| Q2 (0.77-1.33) | 2.69 (0.48-15.10) | 0.71 (0.19-2.66) | 0.48 | Q2 (0.31-0.34) | 1.19 (0.33-4.29) | 0.54 (0.16-1.78) | 0.62 |
| Q3 (1.34-3.11) | 2.54 (0.30-21.66) | 2.31 (0.68-7.83) | 0.95 | Q3 (0.35-0.42) | 0.53 (0.02-12.99) | 1.22 (0.41-3.59) | 0.60 |
| Q4 (3.12-4057.21) | 4.54 (0.86-23.89) | 1.19 (0.35-4.03) | 0.48 | Q4 (0.43-5.58) | 0.88 (0.23-3.31) | 1.26 (0.41-3.92) | 0.80 |
| **Mercury (ng/g)** |  |  |  | **Selenium (μg/g)** |  |  |  |
| Q1 (0.56-3.66) | 1.00 (reference) | 1.00 (reference) |  | Q1 (0.44-0.77) | 1.00 (reference) | 1.00 (reference) |  |
| Q2 (3.66-7.74) | 0.80 (0.26-2.45) | 1.40 (0.44-4.45) | 0.71 | Q2 (0.78-0.86) | 0.61 (0.02-16.62) | 1.23 (0.38-3.97) | 0.65 |
| Q3 (7.75-17.65) | 1.02 (0.26-4.06) | 1.14 (0.32-4.11) | 0.94 | Q3 (0.87-0.97) | 1.26 (0.13-12.60) | 0.73 (0.13-4.01) | 0.72 |
| Q4 (17.76-174.11) | 0.65 (0.02-17.08) | 0.92 (0.28-3.03) | 0.81 | Q4 (0.98-1.98) | 0.35 (0.04-3.39) | 1.03 (0.29-3.60) | 0.54 |
| **Lead (ng/g)** |  |  |  | **Zinc (μg/g)** |  |  |  |
| Q1 (2.60-9.30) | 1.00 (reference) | 1.00 (reference) |  | Q1 (29.87-52.23) | 1.00 (reference) | 1.00 (reference) |  |
| Q2 (9.31-16.60) | 0.94 (0.14-6.32) | 0.71 (0.19-2.70) | 0.84 | Q2 (52.24-60.30) | 1.02 (0.11-9.74) | 0.45 (0.10-2.16) | 0.61 |
| Q3 (16.61-31.60) | 1.71 (0.53-5.52) | 0.58 (0.13-2.65) | 0.53 | Q3 (60.31-71.70) | 0.62 (0.03-14.72) | 1.12 (0.30-4.15) | 0.70 |
| Q4 (31.61-893.10) | 1.76 (0.25-12.39) | 1.00 (0.29-3.40) | 0.71 | Q4 (71.71-487.50) | 1.63 (0.28-9.58) | 0.84 (0.18-3.96) | 0.66 |
| Hazard ratios (95% CI) for neonatal bacterial sepsis by umbilical cord tissue trace element concentration quartiles as estimated by separate discrete-time hazard regression models adjusted for geographic region (Midwest, New England, North Carolina), maternal race/ethnicity (Hispanic, non-Hispanic black, non-Hispanic other, non-Hispanic white), age (years), educational attainment (less than high school diploma, high school diploma, college degree), Medicaid coverage (yes or no), pre-pregnancy BMI (kg/m^2^), and smoking status during pregnancy (yes or no). p_heterogeneity_ for effect measure modification by infant sex calculated with two-sample z-tests. | | | | | | | |

| **Supplemental Table 5. Adjusted associations of umbilical cord tissue trace element mixtures with late-onset neonatal bacterial sepsis in the ELGAN study (n=269)** | |
| --- | --- |
| **Trace Element Mixture** | **HR (95% CI)** |
| All Trace Elements | 0.84 (0.56-1.26) |
| Non-Essential Trace Elements | 0.98 (0.70-1.37) |
| Essential Trace Elements | 0.86 (0.56-1.31) |
| Hazard ratios (95% CI) for neonatal bacterial sepsis for umbilical cord tissue trace element mixtures as estimated by quantile g-computation, adjusted for geographic region (Midwest, New England, North Carolina), maternal race/ethnicity (Hispanic, non-Hispanic black, non-Hispanic other, non-Hispanic white), age (years), educational attainment (less than high school diploma, high school diploma, college degree), Medicaid coverage (yes or no), pre-pregnancy BMI (kg/m^2^), and smoking status during pregnancy (yes or no). The non-essential trace element mixture included arsenic, cadmium, mercury, and lead whereas the essential trace element mixture included copper, manganese, selenium, and zinc. | |

| **Supplemental Table 6. Intermediate-adjusted associations of umbilical cord tissue trace element mixtures with neonatal bacterial sepsis in the ELGAN study (n=250*)** | |
| --- | --- |
| **Trace Element Mixture** | **HR (95% CI)** |
| All Trace Elements | 0.81 (0.56-1.16) |
| Non-Essential Trace Elements | 1.18 (0.87-1.60) |
| Essential Trace Elements | 0.68 (0.48-0.97) |
| Hazard ratios (95% CI) for neonatal bacterial sepsis for umbilical cord tissue trace element mixtures as estimated by quantile g-computation, adjusted for geographic region (Midwest, New England, North Carolina), maternal race/ethnicity (Hispanic, non-Hispanic black, non-Hispanic other, non-Hispanic white), age (years), educational attainment (less than high school diploma, high school diploma, college degree), Medicaid coverage (yes or no), pre-pregnancy BMI (kg/m^2^), smoking status during pregnancy (yes or no), gestational age (weeks), birth weight (grams), chorioamnionitis (yes or no), infections during pregnancy (yes or no), infant sex (female or male), and pPROM (yes or no). The non-essential trace element mixture included arsenic, cadmium, mercury, and lead whereas the essential trace element mixture included copper, manganese, selenium, and zinc. *19 participants were missing placental histology to confirm a diagnosis of chorioamnionitis. | |

| **Supplemental Table 7. Adjusted associations of umbilical cord tissue trace element mixtures with neonatal bacterial sepsis by infant sex in the ELGAN study (n=269)** | | | |
| --- | --- | --- | --- |
|  | **Females**  **(n=120)** | **Males**  **(n=149)** |  |
| **Trace Element Mixture** | **HR (95% CI)** | **HR (95% CI)** | **p_heterogeneity_** |
| All Trace Elements | 0.84 (0.40-1.79) | 0.94 (0.60-1.50) | 0.80 |
| Non-Essential Trace Elements | 1.19 (0.60-2.37) | 1.14 (0.75-1.72) | 0.91 |
| Essential Trace Elements | 0.71 (0.31-1.65) | 0.83 (0.52-1.33) | 0.75 |
| Hazard ratios (95% CI) for neonatal bacterial sepsis for umbilical cord tissue trace element mixtures as estimated by quantile g-computation, adjusted for geographic region (Midwest, New England, North Carolina), maternal race/ethnicity (Hispanic, non-Hispanic black, non-Hispanic other, non-Hispanic white), age (years), educational attainment (less than high school diploma, high school diploma, college degree), Medicaid coverage (yes or no), pre-pregnancy BMI (kg/m^2^), and smoking status during pregnancy (yes or no). The non-essential trace element mixture included arsenic, cadmium, mercury, and lead whereas the essential trace element mixture included copper, manganese, selenium, and zinc. p_heterogeneity_ for effect measure modification by infant sex calculated with two-sample z-tests. | | | |
